# The Fusion Protein of CTP-HBcAg18-27-Tapasin Mediates the Apoptosis of CD8^+^T Cells and CD8^+^ T Cell Response in HLA-A2 Transgenic Mice

**DOI:** 10.5812/hepatmon.16161

**Published:** 2014-02-28

**Authors:** Yu-Yan Tang, Zheng-Hao Tang, Yi Zhang, Meng Zhuo, Guo-Qing Zang, Xiao-Hua Chen, Yong-Sheng Yu

**Affiliations:** 1 Department of Infectious Disease, Shanghai Jiao Tong University Affiliated Sixth People’s Hospital, Shanghai, China

**Keywords:** Tapasin, Mice, Transgenic, T-Lymphocytes, Cytotoxic, PI3K/Akt

## Abstract

**Background:**

HBV-specific cytotoxic T lymphocyte (CTL) activity is believed to play a critical role in controlling HBV infection. The phosphatidylinositol 3-kinase (PI3K)/Akt signaling pathway manipulates cell fate decisions in many different cell types by regulating the activity of downstream effectors. We have previously testified that the fusion protein of CTP-HBcAg18-27--Tapasin could enter the cytoplasm of dendritic cells and efficiently induce robust specific CTL response *in vitro*.

**Objectives:**

In the present study, we evaluated specific CTL response and the level of apoptosis of CD8+ T cells induced by CTP-HBcAg18-27-Tapasin in HLA-A2 transgenic mice (H-2Kb). Meanwhile, we preliminary investigated PI3K, phosphorylation level of Akt, and mammalian target of rapamycin (mTOR) as positive regulator of the magnitude and effector function of the hepatitis B virus-specific cytotoxic T lymphocytes in HLA-A2 transgenic mice.

**Materials and Methods:**

HLA-A2 transgenic mice were immunized by intramuscular injection in the hind legs three times at one-week intervals with PBS, CTP-HBcAg18-27-Tapasin (50 μg), CTP-HBcAg18-27 (50 μg), HBcAg18-27-Tapasin (50 μg), and HBcAg18-27 (50 μg). One week after the last immunization, mice were sacrificed and splenocytes were harvested in strile condition. The specific CTL response was analyzed by flow cytometry and enzyme linked immunosorbent assay (ELISA); the expression of (PI3K)/Akt signaling was detected by RT-PCR and western blot.

**Results:**

The results showed that CTP-HBcAg18-27-Tapasin significantly increased the percentages of IFN-γ^+^ CD8α^+^ T cells, the numbers of these polyfunctional triple-cytokine-producing (IFN-γ, TNF-α, and IL-2) CD8^+^T cells, the secretion of cytokine IFN-γ, IL-2, and TNF-α, while in comparison to control group, it significantly decreased the percentage of apoptotic CD8^+^ T cells in HLA-A2 transgenic mice. Moreover, the expression of PI3K, P-Akt, and P-mTOR was significantly upregulated in CTP-HBcAg18-27-Tapasin group compared with control groups.

**Conclusions:**

In conclusion, CTP-HBcAg18-27-Tapasin could reduce apoptosis of CD8^+^ T cells, increase the percentages of IFN-γ^+^ CD8α^+^ T cells, and elicit cell-mediated immunity in HLA-A2 transgenic mice; these processes were associated with activation of the PI3K/Akt signaling pathway.

## 1. Background

Studies of chronic infections with viruses such as hepatitis B, hepatitis C, and HIV indicate that persistent antigen stimulation induces peripheral T cell tolerance; virus-specific cytotoxic T lymphocyte (CTL) either suffer clonal deletion or lose their functions, a condition termed immunologic tolerance (1, 2). Common denominator underlying antigenic stimulation persistence in these chronic B virus infections (CHB) is the dysregulation of virus-specific T cell responses (3-5). During CHB, the abundance of virus-specific CD8^+^ T cells is controlled by the balance between these cellular processes that a continuum of T cell proliferation and apoptosis (6-8). However, HBV-specific cytotoxic T lymphocyte (CTL) activity might play an important role in HBV clearance, because the magnitude of the CD8^+^ T cell response has a key role in determining the efficiency of viral control (7).

HBV core 18-27 peptide (HBcAg18-27) is recognized as the most efficient agent that primes the human leukocyte antigen (HLA) class-I-restricted immune response in acutely infected patients (9, 10). The HLA-A2 transgenic mice used in the experiments express heterodimeric HLA-A2.1/Kb molecules in the context of a background of H-2 class I molecules (11). HBcAg18-27 is also immunodominant in the context of HLA-A2.1. Previous studies suggest that Tapasin, an endoplasmic reticulum (ER) chaperone, stabilizes the peptide-receptive MHC I conformation, allowing peptide exchange and greater peptide translocation into the ER, which enhances specific MHC class I-restricted CTL activity (12-14). Thus, combining the specificity of CTL epitope (HBcAg18-27), chaperone Tapasin, and transfer by the cell-penetrating property of cytoplasmic transduction peptide (CTP), may elicit a robust specific CTLs response. We have previously testified that the fusion protein CTP-HBcAg18-27-Tapasin could enter the cytoplasm of dendritic cells, and efficiently induce robust specific CTL response, *in vitro* (15, 16).

Mammalian target of rapamycin (mTOR) is a key intermediary in multiple mitogenic signaling pathways and plays a central role in modulating proliferation and angiogenesis in normal tissues and neoplastic processes (17). The PI3K pathway translates numerous extracellular stimuli into a wide range of essential cellular processes through 3-phosphoinositide-dependent effectors such as the serine/threonine kinase Akt. Some Studies previously reported that PI3K is strongly activated in naive T cells after Ag recognition (18-21). During CHB, the abundance of virus-specific CD8^+^ T cells is controlled by the balance between these cellular processes that a continuum of T cell proliferation and apoptosis (6-8). Thus, the PI3K/Akt signaling pathway might be involved in polarization towards CD8^+^ T cells.

## 2. Objectives

In the present study, we evaluated specific CTL response and the level of apoptosis of CD8^+^ T cells induced by CTP-HBcAg18-27-Tapasin in HLA-A2 transgenic mice (H-2Kb). Meanwhile, we preliminary investigated the PI3K, phosphorylation level of Akt, and mammalian target of rapamycin (mTOR) as positive regulators of the magnitude and effector function of the hepatitis B virus-specific CTLs in HLA-A2 transgenic mice.

## 3. Materials and Methods

### 3.1. Reagents, Mice and Fusion Proteins

The fluorescent antibodies and the corresponding isotype controls were obtained from eBioscience (USA), and western blot antibodies were purchased from Abcam (Hong Kong). ELISA kits for IFN-γ, TNF-α, and IL-2 was obtained from R&D Co. Ltd. (USA). Ionomycin, monensin, and phorbol 12-myristate 13-acetate (PMA) were purchased from Sigma (USA). Soluble fusion proteins CTP-HBcAg18-27-Tapasin, CTP-HBcAg18-27, HBcAg18-27-Tapasin, and HBcAg18-27 were maintained in our lab (16).

### 3.2. Mice and Treatments

HLA-A2 transgenic mice (H-2Kb), six to eight weeks old, which had the murine β2 microglo-bulin (β2m), H-2Db genes knocked out, and were transgenic for a chimeric human HLA-A2.1 expressing the a1 and a2 domains of HLA-A2.1 and a mouse H-2Db-derived a3 domain to allow interaction with mouse CD8 (11), were purchased from The Jackson Laboratories and were maintained in the Shanghai Sixth People’s Hospital Animal Centre under specific pathogen-free conditions. All experimental procedures were performed in accordance with approved protocols and regulations by the laboratory animal ethical commission of Shanghai Jiao Tong University. HLA-A2 transgenic mice were allocated into five groups with six mice in each group. Mice were immunized by intramuscular injection of PBS, CTP-HBcAg18-27-Tapasin (50 μg), CTP-HBcAg18-27 (50 μg), HBcAg18-27-Tapasin (50 μg), and HBcAg18-27 (50 μg) in the hind legs three times at one-week intervals. In our preliminary study, we also used the doses of 20μg and 100μg. We found that the dose of 50 μg was the most appropriate dose for our purpose (data not shown). One week after the last immunization, mice were sacrificed and splenocytes were harvested for this experiment in aseptic condition.

### 3.3. Cell Isolation

HLA-A2 transgenic splenocytes were collected and treated with lysis buffer to eliminate red blood cells, washed, and re-suspended in RPMI-1640 (Giboco BRL) with 10% FBS (Giboco BRL). Lymphocytes were derived from splenocytes using nylon wool columns (Wako, Japan). Single-cell suspensions of lymphocytes (2 × 10^6^ cells/well) were grown in six-well plates (Corning). The purities of the isolated T cells were determined by flow cytometry analysis after staining with anti-CD3- PE-Cy5 (eBioscience, United States), and the samples with purity of more than 80% were used for this experiment.

### 3.4. Measurement of Function of CD8+T Cells by Intracellular Cytokine Staining (ICCS)

To investigate the number of IFN-γ secreting cells and also production of TNF-α and IL-2 by the immunized mouse T cells, T lymphocytes (1 × 10^6^ cells/mL) collected from immunized mice were analyzed by flow cytometry. The T lymphocytes were stimulated in the presence of 10 μg/mL HBcAg18-27 for six hours. After incubation for three hours, ionomycin (1 μg/mL), monensin (1.7 μg/mL), and PMA (25 μg/mL) (15) were added and incubation continued for another three hours. After incubation, the wells were washed twice with PBS; cells were then incubated with saturating concentrations of PE conjugated anti-CD8α McAb. After permeabilization with Fix and Perm reagent A and B (BD Biosciences, USA), the cells was stained with FITC-labeled anti-interferon-γ (IFN-γ) McAb, APC conjugated anti-IL-2 McAb, and PE-CY7- labeled anti-TNF-α for 20 minutes. After two washes, the cells were analyzed by flow cytometry (COULTER EPICS XL Flow Cytometer (Beckman)).

### 3.5. Cytokines Release Assay

T cells (2 × 10^6^ cells/mL) from the HLA-A2 transgenic mice harvested from immunized mice were incubated in 24-well plates at 37 °C in the presence of 10 μg/mL HBcAg18-27. After 72 hours of incubation, culture supernatants were harvested and the level of cytokines including IFN-γ, TNF-α and IL-2 were analyzed by ELISA kits according to the manufacturer’s protocol. The concentrations of cytokines in the samples were determined from the standard curves. Data are expressed as pg/mL.

### 3.6. Assessment of Apoptosis Ex Vivo

T cells (2 × 10^6^ cells/mL) from harvested spleens of immunized mice were cultured in six-well plates at 37 °C as described above, except that no red blood cell lysis was performed. After two washes with PBS, cells were incubated with APC-labeled anti-CD8α McAb. Annexin V–FITC and Propidium Iodide (PI) staining (Invitrogen, USA) were then performed according to the manufacturer’s instructions. The whole cell population of thrice stained positive cells among antigen-specific CD8+ T cells was analyzed by flow cytometry.

### 3.7. Real-Time PCR

T cells (2 × 10^6^ cells/mL) from spleens harvested from immunized mice were cultured in six-well plates at 37 °C. Next, cells were collected for total RNA isolation according to the protocol for Trizol Reagent (Invitrogen, USA). cDNA was generated using PrimeScript 1^st^ Strand cDNA Synthesis Kit (TaKaRa, Japan). Primers were designed by Primer Premier 5.0 according to the mRNA sequences of PI3K, Akt, and mTOR genes retrieved from GenBank, and synthesized by Sangon Biotech (Shanghai) Co., Ltd., China. The Primer sequences are shown in Table 1. Real-time PCR was performed using SYBR®Premix Ex TaqTM reagents (TaKaRa, Japan) on a LightCycler (Roche Diagnostic). PCR conditions were as follows: the thermal cycle parameters were 30 seconds at 95 °C followed by 40 cycles of 95 °C for five seconds and 60 °C for 20 seconds. The amount of target was calculated by the following equation: 2^-ΔΔCt^. Three parallel reactions of each sample and internal control were performed.

**Table 1.** The Primer Sequences for PI3K, Akt, mTOR, and β-ctin

Gene	Sequence (5’ to 3’)
**PI3K**	
Forward	TCGGTCTGTAGATGAGGC
Reverse	CGGAGGAATGGATGAGGG
**Akt**	
Forward	G TCGTCGCCAAGGATGAGG
Reverse	GGTCGTGGGTCTGGAATGA
**mTOR**	
Forward	GCCACCTGGTATGAGAAGC
Reverse	CCAACACTGCCCTGTAAAA
**β-ctin**	
Forward	CTCCATCCTGGCCTCGCTCG
Reverse	GCTGTCACCTTCACCGTTCC

### 3.8. Western Blot

The cells described above were washed twice with PBS, gently dispersed into a single-cell suspension, and homogenised using RIPA lysis buffer (Beyotime Institute of Biotechnology, China). Protein concentrations were determined using the Pierce BCA Protein Assay Reagent kit (Rockford, United States). Homogenates were diluted to the desired protein concentration with 2 × SDS-PAGE loading buffer (Invitrogen). Samples were boiled and loaded onto the polyacrylamide mini-gels (Invitrogen) for electrophoresis. Proteins from the gels were transferred to Immobilon-PVDF membranes (Millipore Corp., Bedford, MA, USA) using a semi-dry apparatus (Bio-Rad, Hercules, CA, United States). A rabbit anti-mouse PI3K (1:1000), P-Akt (1:5000), and P-mTOR (1:1000) monoclonal antibody was used as the primary antibody and horseradish peroxidase-conjugated goat anti-rabbit immunoglobulin-G antibody was used as the secondary antibody. Values obtained were normalized based on density values of internal b-actin.

### 3.9. Statistical Analysis

Data were expressed as mean±SD and were analyzed by the SPSS v.16.0 software. One-way ANOVA and post-hoc least significant difference (LSD) test were used to determine the statistical significance in comparison to the control. P-values of 0.05 or less were considered statistically significant.

## 4. Results

### 4.1. CTP-HBcAg18-27-Tapasin Induces IFN-γ-Producing CD8+ T Cells in the Spleen

**Figure 1.**
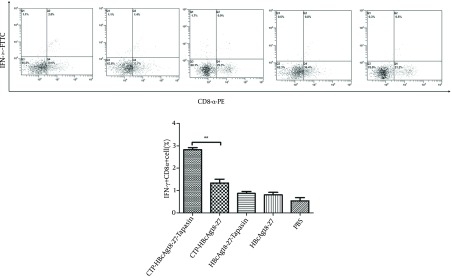
The Percentages of IFN-γ-Producing CD8^+^ T Cells Induced by CTP-HBcAg18-27-Tapasin The whole cell population was analyzed by flow cytometry. CTP-HBcAg18-27-Tapasin enhanced a higher level of HBV-specific IFN-γ^+^ CD8^+^ T cells when compared to CTP-HBcAg18-27, HBcAg18-27-Tapasin, HBcAg18-27, and PBS. The data are presented as mean ± SD from six mice from each group (**P < 0.01).

We measured the amount of IFN-γ-producing CD8^+^ T cells by flow cytometry. The doubly stained cells were the positive ones. As shown in Figure 1, the percentages of specific IFN-γ^+^ CD8^+^ T cells from CTP-HBcAg18-27-Tapasin group (2.83 ± 0.15%) were significantly higher than the percentage of CTP-HBcAg18-27 (1.33 ± 0.31%), HBcAg18-27-Tapasin (0.87 ± 0.15 %), HBcAg18-27 (0.80 ± 0.2 %), and PBS (0.53 ± 0.25 %) (P < 0.01). The results demonstrated that the delivery of Tapasin and HBcAg18-27 via CTP enhanced the generation of IFN-γ^+^CD8^+^ T cells *in vivo*.

### 4.2. CTP-HBcAg18-27-Tapasin Enhances CD8+T Cell Function

**Figure 2.**
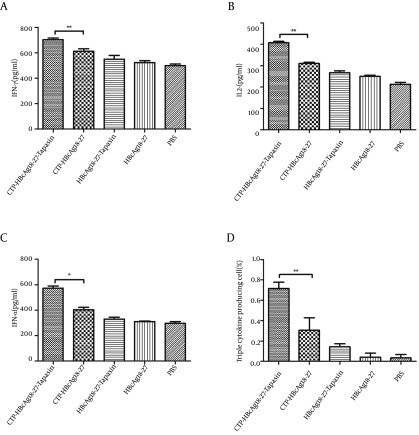
Cytokines Production in the Supernatant of T Cells and Triple-Cytokine-Production IFN-γ, TNF-α, and IL-2 in CD8^+^ T cells. A, B, and C demonstrate that secretions of IFN-γ, TNF-α, and IL-2 in the CTP-HBcAg18-27-Tapasin group were significantly higher than in the CTP-HBcAg18-27, HBcAg18-27-Tapasin, HBcAg18-27, or PBS groups. (D) The numbers of these polyfunctional triple-cytokine-producing (IFN-γ, TNF-α, and IL-2) CD8^+^ T cells in CTP-HBcAg18-27-Tapasin group was higher than the control group. Data represent the mean ± SD (n = 6) (*P < 0.05, **P < 0.01).

Next, we investigated whether the fusion protein of CTP-HBcAg18-27-Tapasin affected the effector function of CD8^+^ T cells. For this purpose, we used ELISA kits and ICCS to measure fusion protein induced production of cytokines (IFN-γ, TNF-α, and IL-2). As shown in Figure 2 A, B, and C, the number of IFN-γ (703.44 ± 21.01 pg/mL), TNF-α (572.82 ± 30.25 pg/mL), and IL-2 (407.34 ± 11.46 pg/mL) production were significantly greater in CTP-HBcAg18-27-Tapasin group than in the CTP-HBcAg18-27 (612 ± 32.45, 310.51 ± 9.85, and 403.63 ± 32.25 pg/mL for IFN-γ, TNF-α and IL-2, respectively), HBcAg18-27-Tapasin, HBcAg18-27, and PBS groups. Notably, the numbers of these polyfunctional triple-cytokine-producing (IFN-γ, TNF-α, and IL-2) CD8^+^ T cells in the CTP-HBcAg18-27-Tapasin group (0.72 ± 0.10 %) was higher than the control groups (Figure 2 D). The inability of CD8^+^ T cells to produce three cytokines is a hallmark of functional exhaustion (22, 23). Thus, our finding suggested that CTP-HBcAg18-27-Tapasin would enhance cytokine IFN-γ, TNF-α, and IL-2 secretion, CD8^+^ T cell function, and elicit cell-mediated immunity.

### 4.3. Decreased Apoptosis of CD8+ T Cells Pulsed With CTP-HBcAg18-27-Tapasin

**Figure 3.**
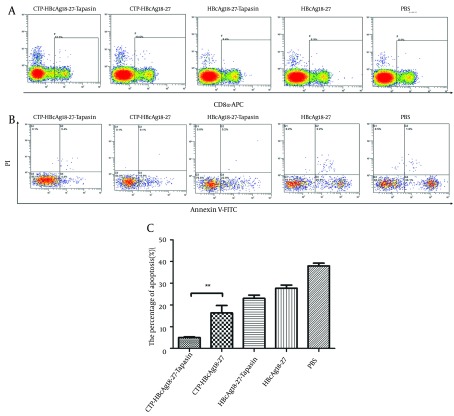
The Apoptosis of CD8^+^ T Cells in T Cells Analyzed by Flow Cytometry The whole cell population was stained three times with fluorescent material labeled using CD8α-APC antibody (A), Annexin V-FITC, and PI (B), and then counted and analyzed by flow cytometry. Significant lower percentages of apoptotic CD8^+^ T cells were observed in mice immunized with CTP-HBcAg18-27-Tapasin. The data are the mean ± SD from six mice per group (**P < 0.01).

The above results indicate that HBcAg18-27 via CTP transduction could efficiently induce CD8^+^ T cell response. However, the mechanism behind these results was not clear. During CHB, the abundance of virus-specific CD8^+^ T cells is controlled by the balance between these cellular processes, resulting in a continuum of T cell proliferation and apoptosis (6-8). Therefore, we further observed the level of apoptosis of CD8^+^ T cells by flow cytometry. The number of three stained positive cells was counted by flow cytometry. As shown in Figure 3, significantly lower percentages of apoptosis of CD8^+^ T cells were observed in mice immunized with CTP-HBcAg18-27-Tapasin (5.01 ± 0.56 %), compared to CTP-HBcAg18-27 (16.30 ± 5.96%), HBcAg18-27-Tapasin (23 ± 2.62%), HBcAg18-27 (27.75 ± 2.40%), and PBS (37.98 ± 2.20 %) (P < 0.01).

### 4.4. CTP-HBcAg18-27-Tapasin Enhanced the CD8+T Cell Response Through Regulating Phosphatidylinositol 3-kinase (PI3K)/Akt Signaling Pathway

**Figure 4.**
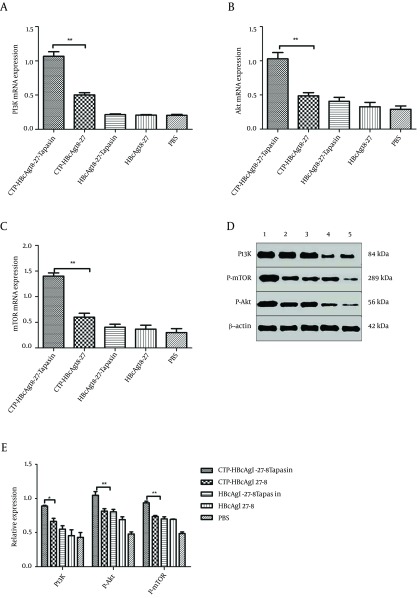
Real-Time PCR and Western Blot Analysis (A, B, C) The expression of PI3K, Akt, and mTOR mRNA were examined by Real-Time PCR. The above expressions were significantly upregulated in CTP-HBcAg18-27-Tapasin group compared with PBS, CTP-HBcAg18-27, HBcAg18-27-Tapasin, and HBcAg18-27 groups. (D, E) Expression of PI3K, P-Akt, and P-mTOR were analyzed by Western blotting. The above proteins expressions were significantly upregulated in CTP-HBcAg18-27-Tapasin group compared with the control groups. 1, CTP-HBcAg18 – 27-Tapasin; 2, CTP-HBcAg18-27; 3, HBcAg18-27-Tapasin; 4, HBcAg18-27; 5, PBS. Data represent the mean ± SD (n = 6) (*P < 0.05, **P < 0.01).

The above results suggested that CTP-HBcAg18-27-Tapasin would decrease apoptosis of CD8^+^ T cells. Next, we investigated the activity of PI3K/Akt signaling pathway in all groups. We further analyzed the PI3K, mTOR, and Akt expression in different groups *in vitro*. The expression of PI3K，mTOR, and Akt mRNA were detected by RT-PCR and the phosphorylation proteins were detected by western blot. The results revealed that expression of PI3K, mTOR, Akt mRNA, and PI3K P-Akt and P-mTOR proteins were significantly upregulated in CTP-HBcAg18-27-Tapasin group in comparison to CTP-HBcAg18-27, HbcAg18-27-Tapasin, HbcAg18-27, and PBS groups (Figure 4). 

## 5. Discussion

Antigen-based immune therapy (vaccine therapy) has emerged as a potential therapeutic approach for CHB patients, as it is based on the concept of viral persistence during HBV infection, it is an inadequate antiviral immune response to the viral antigens (24, 25). The HBV-specific CD8^+^ T cell response plays an important role in the process of HBV clearance (26). Therefore, induction of CTL responses specific to HBV represents a promising strategy to protect against HBV infection. HBV core 18-27 peptide is recognized as the most efficient agent that primes the human leukocyte antigen (HLA) class-I-restricted immune response in acutely infected patients (10). The stable assembly of the MHC class I molecules with peptides is controlled by a number of cofactors, including the peptide-loading complex. Within the peptide-loading complex, the Tapasin is a transmembrane protein that tethers empty class I molecules in the endoplasmic reticulum to the transporter associated with antigen processing, which could promote the surface expression of class I molecule and therefore improve the effectiveness of presentation of peptides to CTLs (27). In addition, it has been demonstrated that the cell-penetrating property of cytoplasmic transduction peptide (CTP) allows it to enter cells when combined with exogenous antigens and induce specific CTL responses (28-30). Thus, combining the specificity of CTL epitope (HBcAg18-27), CTP, and chaperone Tapasin may elicit robust specific HBV immune responses.

We have previously testified that the fusion protein of CTP-HBcAg18-27-Tapasin could enter cytoplasm of dendritic cells, and efficiently induce robust specific CTL response *in vitro* (13). In the present study, we evaluated specific CTL immune responses and the level of apoptosis of CD8^+^ T cells induced by CTP-HBcAg18-27-Tapasin fusion protein in HLA-A2 transgenic mice. At one week after the last immunization of HLA-A2 transgenic mice, the specific IFN-γ^+^ CD8^+^ T cells from CTP-HBcAg18-27-Tapasin group were significantly higher than CTP-HBcAg18-27, HBcAg18-27-Tapasin, HBcAg18-27, and PBS groups, which suggested that the modification of Tapasin would enhance the presentation of target antigens via intracellular delivery to CD8^+^ T cells, and induce stronger cellular immune responses. Furthermore, CTP-HBcAg18-27-Tapasin also enhanced CD8^+^ T cell activity to produce the cytokine IFN-γ, TNF-α, and IL-2. Furthermore, the numbers of these polyfunctional triple-cytokine-producing (IFN-γ, TNF-α, and IL-2) CD8^+^ T cells in CTP-HBcAg18-27-Tapasin group was higher than the control group. The inability of CD8^+^ T cell to produce three cytokines is a hallmark of functional exhaustion (22, 23). This result was consistent with the result of the intracellular expression of IFN-γ in CD8^+^ T cells analyzed by flow cytometry. Taken together, these results indicated that the CTP-HBcAg18-27-Tapasin fusion protein would induce specific CTL responses.

The above results indicated that HBcAg18-27 via CTP transduction would efficiently induce CD8^+^ T cell response. However, the mechanism was not clear. During CHB, the abundance of virus-specific CD8^+^ T cells is controlled by the balance between these cellular processes that results in a continuum of T cell proliferation and apoptosis (6-8). Therefore, we further observed the level of apoptosis of CD8^+^ T cells by flow cytometry. Significant lower percentages of apoptotic CD8^+^ T cells were observed in mice immunized with CTP-HBcAg18-27-Tapasin. This result indicated that CTP-HBcAg18-27-Tapasin could promote CD8^+^ T cell proliferation, which was consistent with the above results. The results showed that CTP-HBcAg18-27-Tapasin would enhance the capacity of CD8^+^ T cells proliferation, cytokines release, and CTLs generation *in vivo*, which could efficiently activate cell-mediated immunity. Although we did not determine HBV specific CTL responses, our study showed that the enhancement of immune responses in the HLA-A2 transgenic mice induced by CTP-HBcAg18-27-Tapasin had several important effects. They included significant increases of the percentages of IFN-γ producing CD8^+^ T cells, and the numbers of these polyfunctional triple-cytokine-producing (IFN-γ, TNF-α, and IL-2) CD8^+^ T cells in the spleen, the secretion of cytokine IFN-γ, IL-2, and TNF-α; on the other hand, it significantly lowered the percentages of apoptotic CD8^+^T cells. These results suggest that the acquisition of the immune responses benefits from combination of the specificity of HBcAg18-27 CTL epitope and Tapasin, and the facilitated delivery of antigens by CTP.

The phosphatidylinositol 3-kinase (PI3K)/Akt kinase-signaling axis plays an important role in a variety of cellular processes, including cytoskeletal dynamics and migration as well as survival and proliferation. For this reason, the pathway is targeted by many pathogens to reinforce or destroy focal adhesions that play an integral role in phagocytosis (31). Some studies have previously reported that PI3K is strongly activated in naive T cells after Ag recognition (21). During CHB, the abundance of virus-specific CD8^+^ T cells is controlled by the balance between these cellular processes that result in a continuum of T cell proliferation and apoptosis (6-8). Thus, the PI3K/Akt signaling pathway might be involved in polarization toward CD8^+^ T cells. In the present study, we further analyzed the PI3K，mTOR, Akt mRNA, PI3K, P-Akt, and P-mTOR proteins expression in different groups. The results revealed that expression of PI3K，mTOR, Akt mRNA, and PI3K P-Akt and P-mTOR proteins were significantly upregulated in CTP-HBcAg18-27-Tapasin group compared with CTP-HBcAg18-27, HBcAg18-27-Tapasin, HBcAg18-27, and PBS group. This result indicated that the CTP-HBcAg18-27-Tapasin fusion protein would induce the pro-survival activity of PI3K-Akt pathway in T cells; this was consistent with the result of the level of apoptosis of CD8^+^ T cells analyzed by flow cytometry. Therefore, the results suggested that this specific CTL activity induced by CTP-HBcAg18-27-Tapasin was related to the activity of PI3K/Akt signaling pathway in HLA-A2 transgenic mice. In conclusion, our results demonstrated that vaccination with soluble CTP-HBcAg18-27-Tapasin fusion protein would reduce apoptosis of CD8^+^ T cells, enhance the CD8^+^T cell response, and elicit cell-mediated immunity in HLA-A2 transgenic mice, which were associated with activation of the PI3K/Akt signaling pathway.
